# Metastasis of Castration-Resistant Prostate Adenocarcinoma to the Lacrimal Gland: A Case Report

**DOI:** 10.3390/reports9010067

**Published:** 2026-02-20

**Authors:** Nikola Milic, Marija Varnicic Lojanica, Milica Ivanovic, Maja Matijasevic, Stefan Ivanovic

**Affiliations:** 1 Užice General Hospital, 31000 Užice, Serbia; 2 Clinic of Obstetrics and Gynecology “Narodni front”, Faculty of Medicine, University of Belgrade, 11000 Belgrade, Serbia

**Keywords:** prostate carcinoma, metastases, lacrimal gland

## Abstract

**Background and Clinical Significance:** Metastases of prostate adenocarcinoma most commonly involve the skeletal system, while orbital metastases, including those in the lacrimal gland, are extremely rare. Despite significant advances in the sensitivity of available imaging methods, their diagnosis is often delayed due to nonspecific clinical presentation and rarity of occurrence. Although exceedingly uncommon, orbital metastases have also been reported in other solid tumors, including certain gynecologic malignancies. **Case presentation:** A 49-year-old patient treated at our center for prostate adenocarcinoma with a Gleason score of 9, regional lymphadenopathy and bone metastases presented to the outpatient clinic with ptosis of the left eyelid, which had developed 7 days prior to examination (13 months after diagnosis of PC). Radiological diagnostics, including CT of the endocranium, revealed enlargement of the left lacrimal gland. An exploratory anterior orbitotomy was performed with an incisional biopsy of the tumor change under retrobulbar anesthesia, and histopathological analysis confirmed a metastatic tumor of the lacrimal gland originating from prostate adenocarcinoma. Despite the application of all available therapeutic measures, a fatal outcome occurred 6 months after the onset of ophthalmic symptoms. **Discussion:** Orbital involvement in metastatic prostate cancer remains poorly characterized due to its extreme rarity and nonspecific clinical presentation. This case emphasizes the need for a high index of suspicion for metastatic disease in patients with known advanced prostate cancer presenting with new orbital or lacrimal gland lesions, as imaging findings alone may be insufficient to distinguish metastases from primary orbital tumors. **Conclusions:** Metastasis of prostate adenocarcinoma to the lacrimal gland is an extremely rare clinical manifestation. Timely diagnosis and adequate radiological assessment are crucial for patient management and survival. The aims of this case report areto present a rare metastatic manifestation of prostate adenocarcinoma with orbital/lacrimal metastasis of prostate adenocarcinoma origin, and to highlight metastatic prostate disease as a potential differential diagnosis in orbital lesions and the importance of imaging methods in their detection.

## 1. Introduction

Prostate cancer (PC) is a common malignant tumor in men, the second most common, right after lung cancer, and the fifth most common cause of death in men worldwide [1]. Genetic and environmental factors play a significant role in its etiopathogenesis, with older age, race, genetic predisposition and positive family history being the main risk factors [2]. Adenocarcinoma occurs in more than 95% of patients with PC [1,2,3]. “Castration-resistant” PC (CRPC) is a form of PC in which disease progression occurs with “castration levels” of serum testosterone [4]. Symptoms are generally absent, so most patients are asymptomatic, especially in the early stages of the disease. Rarely, it can occur as hematuria and/or hematospermia and as nonspecific symptoms of the urogenital tract. In laboratory analyses, elevated values of prostate specific antigen (PSA) in the serum (>4 ng/mL) are specific, but not only for this disease. Physical examination—including digital rectal examination (DRE)—may demonstrate nodules, induration, or asymmetry, which are indications for prostate biopsy. Histological evaluation of prostatic tissue obtained by needle biopsy verifies the diagnosis of PC [3,5]. Tumor staging includes clinical staging (serum PSA values, DRE findings and imaging method findings) and pathological staging (based on tissue analysis and the TNM system). It is important for determining disease severity, assessing prognosis, and recommending therapy [3,6]. Metastatic pathways of PC have not yet been fully elucidated. Lymphogenic metastases occur most often in the para-aortic lymph nodes (LN), and then in the pelvic LN. Hematogenous metastases most often occur in the bones, lungs, liver, pleura and adrenal glands [7]. Orbital metastases, including metastases in the lacrimal gland, are a rare disease and prostate cancer is a rare cause of orbital metastases [8]. Radiological visualization of these lesions can be nonspecific with difficult distinction from primary lacrimal gland tumors [9]. Only sporadic cases of prostate adenocarcinoma metastases to the lacrimal gland are reported in the available literature, which emphasizes the clinical value of each newly documented finding. The aims of this case report areto present a rare metastatic manifestation of prostate adenocarcinoma with orbital/lacrimal metastasis of prostate adenocarcinoma origin, and to highlight metastatic prostate disease as a potential differential diagnosis in orbital lesions and the importance of imaging methods in their detection.

## 2. Case Presentation

**Figure 1:**
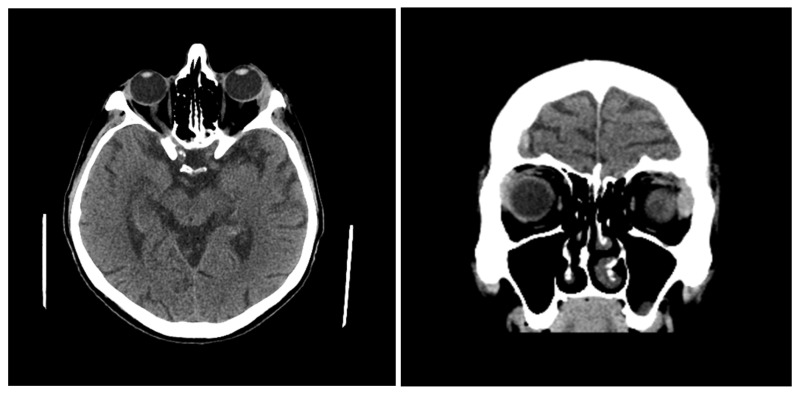
Axial (**left**) and coronal (**right**) CT scans of the endocranium showing enlargement of the left lacrimal gland (17 × 7.7 mm).

We present a 49-year-old patient who was treated for metastatic CRPC with regional lymphadenopathy and bone metastases. Prostate biopsy showed the presence of prostate adenocarcinoma with a Gleason score of 9. The initial value of total PSA (tPSA) in the serum was 26.76 ng/mL and the initial DRE indicated that the prostate was the size of a larger chestnut, limited and painfully sensitive. Initial ultrasonography (US) indicated that the prostate weight was 55 g with prostatolith medially. The initial magnetic resonance imaging (MRI) of the pelvis indicated prostate enlargement with soft tissue tumor change infiltrating the fibrous capsule, periprostatic fat tissue and seminal vesicles. Also, pathologically altered parailiac LNs and bilateral secondary deposits in the pelvic bones were present. Initial skeletal scintigraphy (SS) indicated the presence of signs of increased accumulation of radiopharmaceuticals in the bodies of several thoracic and lumbar spinal vertebrae, the posterior end of the VIII rib on the left side, in the right iliac bone and the distal edge of the sternum. The patient was examined on an outpatient basis by an oncologist, a neurologist and an ophthalmologist at our institution due to the appearance of a drooping eyelid on the left side that appeared 7 days before the examination (13 months after diagnosis of PC). Ptosis of the outer corner of the left eye was described, and other neurological and ophthalmological findings were normal. Personal history was nonspecific (previous ailments and comorbidities were denied); allergies for food or medications and previous surgeries were not reported. The family history was unremarkable. On the same day, a computerized tomography (CT) of the endocranium (Figure 1) was performed in our institution, which indicated an enlargement of the lacrimal gland on the left side (axial diameters 17 × 7.7 mm), as well as the presence of several hyperdense zones/changes on the bones of the calvaria and the base of the skull. These findings differentially and diagnostically corresponded to secondary deposits, and bone depression on the left frontoparietal bone was described. The patient was then sent for examination at a tertiary healthcare institution and a tumor enlargement of the left lacrimal gland was suspected based on the CT findings of the endocranium. An exploratory anterior orbitotomy was performed with an incisional biopsy of the tumor change under retrobulbar anesthesia. The material was sent for pathohistological analysis and a metastatic tumor of the lacrimal gland of prostate adenocarcinoma origin was verified. The operative and postoperative course went well and the local findings were normal at the first follow-up examination. At the next follow-up examination, progression of ptosis of the left eye, upper eyelid swelling and eye pain were described. The patient was presented several times to the Urologic Oncology Council, and initial therapy with LHRH agonists (luteinizing hormone-releasing hormone agonists—leuprorelin) was prescribed with oral administration of bicalutamide and locoregional radiotherapy of affected pelvic bones. There was a progression of the disease (increase in serum PSA levels, progression of clinical symptoms and signs and findings of imaging methods) and the therapy was corrected several times—docetaxel plus prednisone treatment, then therapy with Androgen Receptor Pathway Inhibitor (ARPI) drugs (Abiraterone Acetate), and then cabazitaxel therapy with LHRH agonists. The clinical, diagnostic and therapeutic timeline is presented in Supplementary Materials. Despite the application of all available therapeutic measures, a fatal outcome occurred 6 months after the onset of ophthalmic symptoms.

## 3. Discussion

### 3.1. Castration-Resistant Prostate Cancer and Metastatic Patterns

Adenocarcinoma occurs in more than 95% of patients with PC [3]. “Castration-resistant” PC (CRPC) is a form of PC in which the disease progresses with low serum testosterone levels (up to 50 ng/mL) achieved with androgen deprivation therapy (ADT). According to a literature review, CRPC occurs in 10 to 20% of patients with metastatic PC in the first 5 years of follow-up with a median survival time of about 14 months [4]. It is considered that castration resistance will develop in almost all patients treated with ADT with/or without systemic therapy, regardless of the stage of the disease. There are subtypes of CRPC, such as non-metastatic CRPC and metastatic CRPC [10]. The available literature shows that more than 80% of patients have metastases at the time of CRPC diagnosis and that 33% of patients without metastatic disease at the time of CRPC diagnosis will develop one or more metastases in the next 2 years [11]. Due to the high incidence of CRPC metastatic disease, future studies on the possible correlation between the presence of CRPC and the occurrence of rare metastases could improve the prognosis of these patients. Metastatic pathways of PC have not yet been fully understood. Lymphogenic metastases most often occur in the para-aortic LNs and then in the pelvic LNs [7]. In certain cases, metastases can bypass pelvic LNs and initially appear in common iliac, para-aortic and even supraclavicular LNs [12]. The most common sites of hematogenous metastases are bones (90.1%), namely in the lumbar part of the spinal column (90%) and less often in the thoracic and cervical parts, while they are rare in other bones such as ribs (18.2%), long bones (15%) and bones of the skull (8%) [7]. Bone metastases are most often osteoblastic (66–90%) [13]. Hematogenous metastases occur in the lungs (45.7%), liver (25%), pleura (21%), and adrenal glands (12.8%) [7].

### 3.2. Orbital Metastases of Prostate Cancer

Breast and lung cancers most often give orbital metastases, while prostate cancer participates with only 3% [8]. Orbital metastases are presented by the sudden appearance of symptoms such as proptosis, diplopia, redness, reduction in visual acuity, ptosis, epiphora and pain and are often detected before the diagnosis of the primary tumor. The available literature shows that more than half of the reported cases of orbital metastases of prostate cancer in which orbital symptoms were present were actually the initial manifestation of prostate cancer. Orbital prostate cancer metastases differ from other orbital metastases in that they occur more often in older patients and present as osteoblastic lesions. On the other hand, the majority of orbital tumors present as osteolytic lesions or soft tissue masses [13]. CT most often shows intraconal masses that are well defined and contrast enhancing and osteoblastic lesions. In these patients, the therapy is most often palliative with possible application of radiotherapy, chemotherapy and operative treatment [14,15].

### 3.3. Lacrimal Gland Involvement and Differential Diagnosis

About 10% of expansive lesions in the orbit originate from the lacrimal gland. Metastases rarely occur in the lacrimal gland, and available imaging methods show that their characteristics are similar to primary lacrimal gland tumors [15,16]. Tumors that most often give secondary deposits in the lacrimal gland include breast, prostate, kidney, and thyroid gland cancers as well asmalignant melanoma [9,17,18]. Other metastatic tumors of the orbit may infiltrate the lacrimal gland, such as breast cancer that metastasizes to the soft tissue structures of the orbit, while prostate cancer gives bone metastases [15,19].

### 3.4. Rare Orbital and Lacrimal Metastases in Female Malignancies: Implications for Differential Diagnosis

The presented case represents an extremely rare metastasis of prostatic adenocarcinoma to the lacrimal gland. In the literature, other tumors have also been described that can give rise to orbital or periorbital metastases, which is of significant clinical and diagnostic importance. In recent years, individual case reports of orbital metastases from testicular germ cell tumors, malignant melanoma, and renal carcinoma have been published, which clinically and radiologically presented as tumor masses of the orbit or lacrimal gland [20,21,22,23,24].

Testicular germ cell tumors, particularly choriocarcinoma, are known for their aggressive biological behavior and tendency toward early hematogenous spread, with rare metastases to orbital structures and the lacrimal gland also being described [23]. Malignant melanoma, including desmoplastic and amelanotic variants, may metastasize to the orbit with nonspecific imaging characteristics, often without typical pigmentation, which further complicates the differential diagnosis [24,25,26]. Renal carcinoma also represents a tumor with a marked propensity for atypical metastatic localizations, and orbital metastases may mimic inflammatory or endocrine orbital disorders due to their hypervascularity and clinical presentation [27].

A brief overview of rare orbital metastases in gynecologic malignancies is also provided in order to illustrate overlapping clinical and radiological findings relevant for differential diagnosis across different primary tumor types. Secondary deposits in the orbit and its structures can also occur in gynecological malignancies (endometrial, ovarian, and cervical cancer and gestational trophoblastic disease), but very rarely. Of the gynecological malignancies, choriocarcinoma metastasizes to the central nervous system in approximately 8–15% of cases [17]. By reviewing the literature, we found that deposits in the central nervous system were successfully treated with a combination of neurosurgical, chemotherapeutic and radiotherapy methods. It is interesting that only one case of metastasis of gestational trophoblastic disease to the lacrimal gland was recorded in the world literature, where the patient had presented ophthalmological symptoms and radiological signs of tumor infiltration of the upper-outer part of the orbit [18]. There are also isolated reports of orbital metastases of endometrial cancer and epithelial ovarian cancer (a patient with ovarian cancer had a BRCA2 mutation) [19,20,21]. A case of orbital involvement in squamous cell carcinoma of the cervix is also described. All these metastases of gynecological origin were usually observed in the final stages of the disease [22].

Common to all these entities is that the clinical findings of orbital metastases, due to nonspecific symptoms and often vague radiological presentation, can overlap with benign, inflammatory or endocrine changes of the orbit. Therefore, these diagnoses are easily overlooked. Patients are usually older and present with unilateral or bilateral masses in the region of the lacrimal gland (palpable masses in the superolateral part of the orbit) without specific signs of involvement of the eyeball, and associated bone metastases may be present [9]. Patients may also present inferonasal dislocation of the eyeball, proptosis, ptosis, diplopia, eyeball movement disorders and loss of vision as a result of lacrimal gland enlargement. Periorbital pain may also occur. Biopsy enables pathohistological diagnosis and therapy depending on the type, grade and stage of the malignant tumor [16]. US, CT and MRI are the imaging methods of choice in the diagnosis of lacrimal gland disorders. US is useful in the identification of cystic and solid lesions and allows defining of localization, edges, tissue structure/echogenicity and degree of local infiltration. CT enables a clear visualization of the lacrimal gland (which is similar in density to the muscles), especially due to its position (it is limited by the bony walls of the orbit and hypodense orbital fat tissue) and vascularization (increased vascularity in many inflammatory and neoplastic processes that are more clearly visualized following contrast medium enhancement). Bone structures are well delineated including high sensitivity in the display of calcifications. MRI has a lower diagnostic value in the detection of bone erosions, sclerosis and destruction, but is a superior method in the detection of soft tissue changes. The most common MRI sequences used for imaging of the orbit and lacrimal glands are short spin-echo T1-weighted sequences in the axial and coronal planes, T2-weighted fast spin-echo sequences in the axial plane with fat suppression, and post-Gadolinium axial and coronal T1-weighted sequences with fat suppression. Fat suppression enhances anatomical details and allows the detection of pathological changes that would otherwise be obscured by a high signal return from orbital fat. A normal lacrimal gland shows moderate, sometimes heterogeneous, signal intensity on T1-weighted sequences, and after administration of Gadolinium, it is enhanced similarly to after administration of contrast medium in CT [15]. An overview of reported orbital and lacrimal gland metastases from different primary malignancies relevant for differential diagnosis is summarized in Table 1. The differential diagnosis of lacrimal gland enlargement and orbital masses in oncologic patients is outlined in Table 2.

## 4. Conclusions

Metastases in the orbit and lacrimal gland represent one of the rarest sites of prostate cancer metastases. Their clinical manifestation is similar to other tumors of the lacrimal gland and orbit (primary and secondary), as well as benign, inflammatory or endocrinological diseases, which highlights the importance of imaging methods in their diagnosis. With the advancement of technology, the development of future, more sensitive and specific imaging methods will enable better delineation of these lesions, which can contribute to timely diagnosis and may improve the prognosis and outcomes of these patients. We conclude that neither the domestic nor the international literature offers a large number of such representations, especially when it comes to cases of metastases of the lacrimal gland, which highlights the scientific value of such case reports.

## Figures and Tables

**Table 1:** Reported orbital and lacrimal gland metastases from solid tumors relevant for differential diagnosis.

Primary Malignancy	Orbital/Lacrimal Gland Involvement	Typical Imaging Findings	Histopathological Confirmation	Clinical Context/ Remarks	References
Prostate adenocarcinoma	Orbit, lacrimal gland	Intraconal mass, osteoblastic bone lesions, lacrimal gland enlargement	Adenocarcinoma	Rare; often advanced disease; may mimic primary lacrimal tumors	[8,13,14]
Breast carcinoma	Orbit, lacrimal gland infiltration	Soft tissue mass, bone involvement	Carcinoma metastasis	Most common source of orbital metastases	[8,9,15]
Lung carcinoma	Orbit	Intraconal or extraconal mass, bone destruction	Carcinoma metastasis	Frequent orbital metastases; rapid progression	[8,13]
Renal cell carcinoma	Orbit	Hypervascular orbital mass, bone involvement	Clear cell carcinoma	Can mimic inflammatory or endocrine orbital disease	[27]
Malignant melanoma	Orbit	Infiltrative orbital mass, variable signal intensity	Melanoma metastasis	May be amelanotic; nonspecific imaging	[24,25,26]
Testicular germ cell tumor (choriocarcinoma)	Orbit/lacrimal gland	Orbital soft tissue mass	Choriocarcinoma metastasis	Aggressive tumor with early hematogenous spread	[23]
Endometrial carcinoma	Orbit	Cystic or solid orbital lesion	Carcinoma metastasis	Usually late-stage disease	[19]
Ovarian carcinoma	Orbit	Retroorbital mass, soft tissue involvement	Epithelial ovarian carcinoma	Rare; reported in advanced disease	[20,21]
Cervical carcinoma	Orbit	Orbital mass, bone involvement	Squamous cell carcinoma	Very rare orbital metastasis	[22]
Gestational trophoblastic disease (choriocarcinoma)	Orbit/lacrimal gland	Tumor infiltration of superolateral orbit	Choriocarcinoma	Extremely rare; aggressive hematogenous spread	[17,18]

**Table 2:** Differential diagnosis of lacrimal gland enlargement and orbital masses in oncologic patients.

Etiology	Typical Imaging Features	Clinical Presentation	Key Distinguishing Features	Common Primary Tumors/Conditions
Metastatic disease	Lacrimal gland enlargement, intraconal or extraconal mass, possible bone involvement (osteoblastic or osteolytic)	Rapid onset of ptosis, proptosis, diplopia, pain	History of malignancy; rapid progression; hematogenous spread	Prostate, breast, lung, melanoma, renal cell carcinoma, testicular tumors, gynecologic malignancies
Primary lacrimal gland tumors	Well-defined lacrimal mass, bone remodeling or erosion	Slowly progressive painless swelling	Long-standing symptoms; localized disease	Pleomorphic adenoma, adenoid cystic carcinoma
Inflammatory orbital disease	Diffuse gland enlargement, homogeneous contrast enhancement	Pain, erythema, eyelid edema	Good response to corticosteroids	Idiopathic orbital inflammation, sarcoidosis
Lymphoproliferative disorders	Homogeneous soft tissue mass, molding around structures	Mild pain or painless swelling, often bilateral	Systemic signs; bilateral involvement	Lymphoma, leukemia
Endocrine-related disease	Extraocular muscle enlargement, orbital fat infiltration	Proptosis, lid retraction	Thyroid dysfunction signs	Thyroid eye disease
Infectious processes	Diffuse enlargement, abscess formation (rare)	Pain, fever, inflammatory signs	Laboratory signs of infection	Bacterial or viral orbital infection
Benign cystic lesions	Well-circumscribed cystic lesion	Slowly progressive, painless	No bone destruction; stable size	Dacryops, dermoid cyst

## Data Availability

Data presented in this study are available from the corresponding author upon request.

## Supplementary Materials

The following supporting information can be downloaded at: https://www.mdpi.com/article/10.3390/reports9010067/s1.

## Abbreviations

The following abbreviations are used in this manuscript:

PC	Prostate cancer
CRPC	Castration-resistant prostate cancer
PSA/tPSA	Prostate-specific antigen/Total prostate-specific antigen
DRE	Digital rectal examination
US	Ultrasound/Ultrasonography
MRI	Magnetic resonance imaging
SS	Skeletal scintigraphy
MDCT/CT	Multidetector computed tomography/Computed tomography
LN/LNs	Lymph node(s)
TNM	Tumor–Node–Metastasis classification
ADT	Androgen deprivation therapy
LHRH	Luteinizing hormone-releasing hormone
ARTA	Androgen receptor–targeted agents
