# Supplementary material for: Metastasis of Castration-Resistant Prostate Adenocarcinoma to the Lacrimal Gland: A Case Report
Source: Reports (MDPI). 2026 Feb 20;9(1):67. doi: 10.3390/reports9010067 (PMC13030364; doi:10.3390/reports9010067)
Supplement: Supplementary file 1 [file reports-09-00067-s001.zip › reports-4129868-supplementary.pdf]

Timeline	Clinical presentation, Diagnostic procedures findings and Therapy
**Initial presentation**	**Clinical findings**: urinary urgency, nocturia and low abdomen pain radiating to the scrotum, difficulty walking.<br>**Serum PSA:** 26,76 ng/ml.<br>**Serum testosterone:** /<br>**DRP:** size of a larger chestnut, limited and painfully sensitive.<br>**US:** prostate weight was 55g with prostatolith medially.<br>**Pelvis MR:** prostate enlargement with soft tissue tumor change infiltrating the fibrous capsule, periprostatic fat tissue and seminal vesicles. Also, the pathologically altered parailiac LNs and bilateral secondary deposits in the pelvic bones were presented.<br>**SS:** presence of signs of increased accumulation of radiopharmaceuticals in the bodies of several thoracic and lumbar spinal vertebrae, the posterior edge of the VIII rib on the left side, in the right iliac bone and the distal edge of the sternum.<br>**Therapy**: Antibiotic (Ciprofloxacin) and Alpha-1 blocker (Tamsulosin).
**Initial Urologic Oncology Council (1 month later)**	**Prostate biopsy** – Prostate Adenocarcinoma with a Gleason score of 9.<br>Prostatic adenocarcinoma with regional lymphadenopathy and bone metastases diagnosis was confirmed.<br>**Serum PSA:** 33,97 ng/ml.<br>**DRP:** size of a green walnut, right lobe hard on palpation, prostate not clearly limited on palpation.<br>**US:** prostate was heterogeous, weight was 57g.<br>**SS:** signs of increased accumulation of radiopharmaceuticals in the bodies of Th3, Th5, Th10, L2 and L4 spinal vertebrae, the posterior end of the left rib, right iliac bone and distal border of the sternum.<br>**Therapy:** 6-month LHRH agonist (Leuprorelin) dose with Bicalutamide for 14 days (before and after Leupoprelin dose) was administered; pelvic radiotherapy was recommended; bisphosphonates were recommended.
**Oncologic follow-up (4 monts later)**	**Radical pelvic radiotherapy** was conducted.<br>**Serum PSA:** 8,75 ng/ml.<br>**Serum testosterone:** 0,15 ng/dl.

**Urologic follow-up (7 months later)**	**Serum PSA:** 25,6 g/ml.<br>**Serum testosterone:** 0,15 ng/dl.<br>**US:** prostate clearly limited, weight was 32g.<br>**CT of abdomen and pelvis:** Both kidneys are of regular size, without hydronephrosis. Prostate with a transverse diameter of about 39x35mm. Bone structures with numerous osteosclerotic metastatic leasions - on most vertebral bodies (Th7, Th8, Th 10 with involvement of posterior vertebral elements on the right, Th11, L1, L2, L3, L4, L5, S1, S2), on both sides on the bones of the pelvis, more pronounced on the right side, on the distal part of the sternum, on the back ends of the VIII rib on both sides, in the region of the neck of the right femur. Liver craniocaudal diameter measured 16.5 cm with "Too Small to Characterize" (TSTC) lesion in IV segment, without distinguishing other focal changes.<br>**SS**: progression of number and intensity of accumulation of radiopharmaceuticals in previously described lesions.<br>**Therapy:** Tamsulosin+Dutasterid.
**Oncologic follow-up (7 months later)**	**Therapy:** 6-month Leuprorelin dose with Bicalutamide for 14 days was administered.
**Urologic Oncology Council (8 months later)**	Disease progression and CRPC diagnosis was confirmed.<br>**Therapy:** docetaxel plus prednisone treatment.
**Oncologic follow-up (9 months later)**	First cycle of docetaxel plus prednisone.
**Oncologic follow-up (10 months later)**	Second cycle of docetaxel plus prednisone,<br>later patient was hospitalized in the Oncologic department for correction of moderate anaemia.
**Oncologic follow-up (11 months later)**	Third cycle of docetaxel plus prednisone.
**Oncologic follow-up (12 months later)**	Fourth cycle of docetaxel plus prednisone, later patient was hospitalized in the Oncologic department for correction of moderate anaemia.
**Urologic Oncology Council (13 months later)**	Disease progression (including progression of SS findings)<br>**Therapy:** Androgen Receptor Pathway Inhibitors (ARPI) - Abiraterone Acetate.
**Oncologic follow-up (14 moths later)**	**Clinical findings**: Appearance of a drooping eyelid on the left side that appeared 7 days before the examination; Ptosis of the outer corner of the left eye was described and other neurological and ophthalmological findings were normal.

**Oncologic follow-up (14 moths later)**	**CT of endocranium:** enlargement of the lacrimal gland on the left side (axial diameters 17 x 7.7 mm), the presence of several hyperdense zones/changes on the bones of the calvaria and the base of the skull which differentially and diagnostically corresponded to secondary deposits, and bone depression on the left frontoparietal bone.
**Tertiary Ophthalmic Clinic (15 months later)**	Exploratory anterior orbitotomy with an incisional biopsy of the tumor change under retrobulbar anesthesia.<br>**Pathohistological analysis –** metastatic tumor of lacrimal glad of prostate adenocarcinoma origin.<br>Operative and postoperative course went well and the local findings were normal at the first follow-up.
**Oncologic follow-up (16 months later)**	Patient was hospitalized in the Oncologic department for correction of moderate anaemia and fever.
**Oncologic follow-up (17 months later)**	**Clinical findings**: Abdomial pain, appetite loss and fever one day prior to examination; ptosis, eyelid swelling and eye pain few months prior to examination.<br>**US:** numerous predominantly hyperechoic changes up to 38 mm in diameter in both lobes of the liver that were differentially and diagnostically corresponded to secondary deposits. Prostate was inhomogeneous, with a volume of 30 ml and a pleural effusion was described on the left side. Later patient was hospitalized in the Oncologic department for correction of moderate anaemia.
**Urologic Oncology Council (18 months later)**	Disease progression<br>**Therapy:** 3-month Leupoprelin dose, cabazitaxel
**Urology department (20 months later)**	Hospitalized in the Urologic department for correction of moderate anaemia.<br>**Clinical findings**: weakness, malaise,difficulty breathing, fever, abdominal pain, back pain; ptosis, eyelid swelling and eye pain several months prior to examination.<br>**Chest Radiography:** large pleural effusions on the both sides<br>**US:** large pleural effusions on both sides, numerous, previously described solitary (diameter about 40 mm) and confluent changes towards the left lobe (diameter about 60 mm) in the liver and the presence of free fluid in the abdomen.<br>**Thoracocetesis** was performed twice - first time 1500 ml of was obtained which corresponded to empyema and second time 1000ml of serous fluid was obtained<br>About 20 days after hospitalization there was rapid deterioration of the patients general condition and despite the application of all available therapeutic measures, a fatal outcome occurred.
